# Exploring the Enjoyment of the Intergenerational Physical Activity

**DOI:** 10.3390/jfmk6020051

**Published:** 2021-06-14

**Authors:** Andrea Buonsenso, Giovanni Fiorilli, Cristiana Mosca, Marco Centorbi, Concetta C. Notarstefano, Giulia Di Martino, Giuseppe Calcagno, Mariano Intrieri, Alessandra di Cagno

**Affiliations:** 1 Department of Medicine and Health Sciences, University of Molise, v. De Sanctis 1, 86100 Campobasso, Italy; andreabuonsenso@gmail.com (A.B.); fiorilli@unimol.it (G.F.); marco.centorbi@hotmail.it (M.C.); intrieri@unimol.it (M.I.); 2 Department of Motor, Human and Health Sciences, University of Rome “Foro Italico”, Lauro de Bosis Square, 15, 00197 Rome, Italy; cristiana.mosca1994@gmail.com (C.M.); connythe@hotmail.it (C.C.N.); giulia.dimartino21@gmail.com (G.D.M.); alessandra.dicagno@uniroma4.it (A.d.C.)

**Keywords:** exercise, preschooler, older adults, adherence

## Abstract

Intergenerational physical activity could be a pleasant method to prevent elderly sedentary behaviors. The aim of this study is to provide a basis to develop an intergenerational physical activity between preschool children and elderly people. An assessing enjoyment three questionnaire survey was administered to 140 participants (aged 67.8 ± 9.1): the global physical activity questionnaire (GPAQ) assessing the sedentariness degree; the physical activity enjoyment scale (PACES-Q) assessing enjoyment for the physical activity usually practiced; the physical activity enjoyment scale (PACES-INT) assessing the enjoyment for a hypothetical intergenerational program. Successively, the sample was divided into subgroups based on age, gender, marital status, education, employment, sports background, sedentariness level and residential location. Four multichoice questions, aiming to have guidelines in organizing an intergenerational program, were used. A total of 44.3% of the sample found the physical activity practiced pleasant, whereas 81.5% enjoyed the intergenerational program (only 7.1% expressed a negative judgment). A separated one-way ANOVA showed significant differences in PACES-INT for gender, (*p* = 0.009), residential location, (*p* < 0.001) and employment (*p* = 0.004). About 80% of the sample would adhere to the intergenerational programs, despite the fatigue fear and logistic or family relationship problems.

## 1. Introduction

Sedentariness between children (3 and 5 years of age) and elderly (65 or over) has become a national emergency in Italy. The percentage of elderly sedentary people increases with the advancing age. It reaches 45.1% in people over than 65 y.o. and 69.8% in people over 75 y.o. [1]. Nine potential but modifiable life-style risk factors in elderly have been identified: less education, hypertension, hearing impairment, smoking, obesity, depression, physical inactivity, diabetes and low social contacts [2]. Regular physical activity (PA), especially practices for a prolonged time, positively influences almost all the other risk factors, with minimal side effects [3]. PA helps to improve muscle regenerative capacity [4,5], and to prevent metabolic syndrome [6] and breast cancer [7]. The aerobic PA carried out regularly positively affects cardiorespiratory fitness and, improving the executive functions and memory, may slow down the cognitive decline [8]. The first goal in treatment of frailty is to maintain a person’s physical independence as long as possible [9]. The World Health Organization (WHO) [10] (2010) recommendations for older adults include a moderate to vigorous aerobic PA (150 min/week) or vigorous activity performing muscle strengthening, balance and mobility (75 min min/week).

According to the data of National Institute of Statistics (ISTAT), 46.1% of preschoolers children do not participate in sport at all or are engaged in physical activity during their leisure time [11]. WHO recommendations include moderate to vigorous physical activity (PA) of at least 60 min per day for preschool children [10], and 180 min per day of physical activity at any intensity for children of 3–4 years [12] (WHO, 2020): these goals are not yet met. PA leads to improvements in cognitive development, such as executive functions and language skills [13].

Sedentary behavior is associated with poorer health outcomes, in both the age groups, with impact on lifestyle and wellness: in children, sedentary time, especially spent watching television, is associated with a high risk of adiposity and overweight [14], and negative effects on psychosocial health and lack of motor skills development [15]. In older people, especially in the old-older, the consequences are related to worsening quality of life, functional limitations, pain, anxiety and depression [16].

The intergenerational programs could be a proposal to involve both the two generations in a physical activity program, practiced closer together. These programs were rapidly developed, from the 1960s to the 1970s, meeting the needs of young and older people to recover their social interaction, changed over the time [17]. Since the 2000s the United Nations have promoted the solidarity strengthening between generations, developing some initiatives to this purpose [18]. The solidarity between generations was recognized as a strategy to achieve active ageing. In 2012 the European Union declared “2012 Year of Active Ageing and Solidarity between Generations”and promoted several intergenerational programs [19], such as the project Together Old and Young [20].

In the elderly, intergenerational programs seem to prevent loneliness and reduce depression [21], improve attitudes towards young people and participants self-esteem and generativity [22]. Moreover, these programs effectively maintain physical functioning, intellectual activities, mental well-being and health-related life quality [23].

In young people, intergenerational programs lead to decrease preconceptions and negative attitudes towards elderly, improving in empathy [22], prosocial behavior and the ability to regulate their behaviors [24]. Finally, some evidence seems to be found in school performance, resilience and self-esteem [25].

The aim of this study was to investigate the availability and level of enjoyment in elderly involved in intergenerational PA. A survey on elderly population enjoyment to the intergenerational programs organized between preschool children and people over 65, was conducted amongst Italian older people using validated questionnaires. In Italy, no intergenerational programs, aimed to improve PA and active lifestyle, had been promoted yet.

## 2. Materials and Methods

### 2.1. Study Design

The project proposed a PA program performed by the elderly and children together, in shared distanced spaces and with common goals, entertaining all the participants in an active manner. This condition could increase the level of enjoyment for PA, which is the major factor leading to better adherence to this intergenerational program. To assess the enjoyment degree for this proposal a survey design was used. At the end of the PA program four questions at multiple answers were proposed to assess participants’ opinions and preferences on an eventual shared PA between generations.

### 2.2. Participants

One hundred and forty individuals, aged between 50 and 85 years, volunteered for the study. Fifty participants were recruited from a gentle postural gymnastics group for the elderly, 30 attended a leisure centre for the elderly and a snowball sampling strategy focused on recruiting the remaining participants was used. The only inclusion criteria were to be over 50 years old. A cover letter providing information on the nature of the research, was delivered to all participants. The assurance of confidentiality and anonymity was included.

The personal data were collected anonymously by the creation of a personal security code. Written informed consent on the study purposes and for the data processing was obtained from all participants. The sample characteristics are shown in Table 1. The study was designed and conducted in accordance with the Declaration of Helsinki and approved by the local bioethical committee of the University of Rome “Foro Italico” (CAR—68/2020).

### 2.3. Procedures

The self-administered questionnaires were the global physical activity questionnaire (GPAQ). They were focused to assess the level of PA, by which the sample was divided into active and inactive. The enjoyment for the PA practiced was assessed using the physical activity enjoyment scale (PACES-Q) and the enjoyment for a hypothetical intergenerational PA (PACES-INT). Successively the sample was divided into subgroups based on age, gender, marital status, education, employment, sports background, sedentariness level and residential location, to assess differences amongst groups in their responses.

#### 2.3.1. The Global Physical Activity Questionnaire (GPAQ)

The global physical activity questionnaire (GPAQ) [26] is a validated questionnaire to assess the level of PA of the adult population [27] and it is both easy and quick to compile. It allows for estimating the total sedentary time. The GPAQ is composed of 16-items, two choice answers (yes/no) about PA Level in different moments of the day, and its duration (hours/minutes), if required: the activities carried out during the work (P1–P6), the displacements from one place to another (P7–P9), the recreational activities (P10–P15) and the time employed in sedentary behaviors (P16). The results provided information on the sedentary status. The following equation was used to calculate total physical activity MET min/week: ((P2 × P3 × 8) + (P5 × P6 × 4) + (P8 × P9 × 4) + (P11 × P12 × 8) + (P14 × P15 × 4)). A score of less than 600 classifies the subject as inactive; a score of 600 or more classifies the subject as active.

#### 2.3.2. Physical Activity Enjoyment Scale (PACES-Q)

To assess the subjective degree of enjoyment in carrying out practical activities, the physical activity enjoyment scale (PACES) was used.

The PACES, validated for the Italian version [28] and older people [29], is composed of 8-items with a Likert rating scale from 1 (strongly disagreed) to 5 (strongly agreed). A total score of 21 or less indicates a positive enjoyment; a score between 22 and 26 indicates a neutral consideration; a score of 27 or more indicates a negative enjoyment of physical activity.

#### 2.3.3. Physical Activity Enjoyment Scale for Intergenerational Physical Activity (PACES-INT)

To assess the subjective degree of enjoyment for an intergenerational physical activity, the PACES questionnaire was used, by adding an introductory explanation: “Imagine doing structured physical activity with your nephew or a child between 3 and 5 years. The activity will include games and movements to perform together in shared distanced spaces and with common goals”. The questionnaire was administered and interpreted in the same modalities of PACES-Q.

#### 2.3.4. Multichoice Questions

Four multichoice questions with a three-point scale were added at the end of the questionnaires to obtain guidelines in organizing the project. At the end of the questionnaires, 4 multichoice questions with a three-point scale, aiming to have guidelines in organizing the project, were added.

### 2.4. Statistical Analysis

Data analysis was performed using SPSS ver.23 (IBM, Armonk, NY, USA). The normal distribution of continuous variables was verified through the Kolmogorov–Smirnov test. For continuous variables normally distributed, mean ± SD were reported. Separated one-way analysis of variance (ANOVA) tests was performed to test the differences among the PACES-Q and PACES-INT as dependent variables. The independent variables were age (under 70, over 70), gender, marital status (married, not married), education (elementary and middle school diploma, secondary school and bachelor degree), employment (housewife and pensioners, other job), sports background (former-sportsman, non-sportsman), Sedentariness level (active, inactive) and residential location (rural, urban).

Pearson’s product correlation analysis was performed between age and the level of satisfaction (PACES-Q and PACES-INT). The alpha test level for statistical significance for all variables was set at 0.05.

## 3. Results

### 3.1. GPAQ

The GPAQ analysis divided the sample according to the PA carried out during the week. The analysis showed that 21.4% of the participants were actives (30 subjects) while 78.6% were inactive (110 subjects). Results are shown in Table 1.

### 3.2. PACES-Q

**Figure 1:**
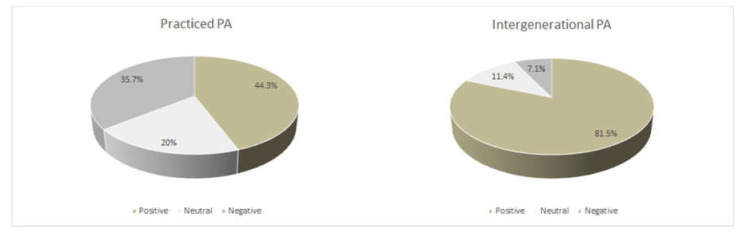
Enjoyment for physical activity.

Separated one-way ANOVA showed no significant differences in PACES-Q for age, gender, education, sport background, residential location, marital status, sedentariness level and employment. The enjoyment results for PA practiced were shown in Figure 1.

The Pearson correlation showed a modest and inverse correlation (r = −0.363) between PACES Q and age (*p* < 0.001).

### 3.3. PACES-INT

Separated one-way ANOVA showed significant differences in PACES-INT for gender, where women reported lower scores than men (*p* = 0.009); for residential location, where the rural group reported lower scores than the urban group (*p* < 0.001), and for employment, where the housewife and pensioners reported lower scores than the other job group (*p* = 0.004). No statistical difference was found for age, education, sports background, marital status and sedentariness level. The enjoyment results for intergenerational PA were shown in Figure 1. Mean ± SD deviation are reported in Table 2. The enjoyment of all subgroups to PACES-Q and PACES-INT is shown in Table 3.

### 3.4. Multichoice Questions

The responses, in percentage, to the multichoice questions are shown in Table 4.

## 4. Discussion

The main result of the present survey was that the level of enjoyment for the intergenerational PA proposal turned out to be positive for the 81.5% of the sample (only 7.1% expresses a negative judgment). It was a relevant result, considering that only 44.3% of the participants declared to find pleasant their PA that they carried out daily or weekly. Participants who had a neutral or negative percentage of enjoyment for their usual PA gave positive feedback on the intergenerational proposal, considering it pleasant. In the present study, it was hypothesized that the acceptance for this new proposal could be due to the original and funny nature of the intergenerational PA, compared to those usually proposed. It is demonstrated that enjoyment promotes adherence to PA programs [30]. Ransdell and colleagues [31] reported that participation in activities with children increased membership and motivated a more active lifestyle. Accordingly, the pastime grandparents’ favorite, which they frequently carry out, might involve the children and influence their level of PA leading to greater participation in PA of the children [32]. The intergenerational program, of this survey, including persons of the same family, might ensure good adherence and therefore guarantee the success of this proposal.

Considering the sample divided between active and non-actives, no significant differences in the level of enjoyment both for their usual practice PA and intergenerational PA were found. Despite that the participants are aware of the protective health benefits of regular PA, the active participants did not consider their practice PA motivating and did not find a potential appeal and engagement in this type of PA [30]. Less than half of the sample who perform regular PA declared to be satisfied with the activity carried out daily or weekly.

According to gender, employment and residential location, significant differences were found in the sample responses. Significant gender-based differences were found on the degree of enjoyment for both the two types of PA. Women were motivated by losing or managing weight and improving appearance through PA than men, and this condition might optimize the potential PA appeal and engagement, independently of age [33]. Moreover, even low levels of PA decrease the risk of psychological diseases such as depression and anxiety in women. Such as motivating factor to “feel good”, PA can improve mood, enhancing social interactions and quality of life [34]. Older women have few opportunities to create social networks and are more likely to be motivated by social factors than men [35] and frequently are engaged with their nephews’ cares. Differences in preferred activities between genders may have motivated the better predisposition of women for intergenerational programs: men are more likely to prefer sports that require vigorous activities, or that involve them in competition and outdoor activities, whereas women have a stronger preference to perform indoor PA [36].

According to the residential location, it emerges that rural inhabitants are more likely to enjoy intergenerational PA than the urban population. People living in rural contexts have greater opportunities to pursue an active lifestyle [37], far from the busy life of the city and consequently, they could be more open to new proposals. Although urban residents tend to follow the PA guidelines, they are less active in the daily life, using transportation means and dedicating less time to occupational and domestic tasks, consequently, they have a greater need for structured PA than rural ones [38]. In the other hand, rural residents are less engaged for a time in high-intensity PA, organized in the gymnasium [39], and are more interested in new PA proposals. Another hypothesized explanation of this result might be that in the urban context it is more difficult reaching different places to pick up the children and go to the sports facilities, than in the rural contexts.

Considering the different employments, results showed that housewives and pensioners significantly enjoyed intergenerational PA more than those who were employed in paid work. The underlying rationale was that, for an employed, performing a weekly PA is related to the perception of “lost time” for their work, adding the difficulty to reach workplace of PA interventions [40]. For the housewives and pensioners, this proposal and generally PA might mean a different and funny use of their time, in contrast with those who, working outside, excluded PA for a lack of time. Moreover, as a previous study highlighted, housewives are influenced by their surroundings for adopting healthy behaviors, thus, employing family members or other important persons of them, in their PA programs could be an effective influencing factor [41]. An active psychosocial environment seems to facilitate also the pension age state [42]. Intergenerational proposal matches with these demands.

No significant differences were found in the sample, divided on marital status, education, and sports background. Although our sample was regionally representative, it might be not large enough to allow these further stratifications.

Marital status did not influence the sample response. Scientific literature, in contrast, showed that married people of older age have higher levels of PA, especially if they have a partner who habitually practices PA [43].

The sample divided for their level of education showed only a response trend that indicated the individuals with low school qualifications as more available with an intergenerational proposal of PA. This is an unexpected trend considering the results of previous studies [44,45]. We hypothesized that graduates’ participants, having a challenging job that may engage them for a lot of time, are more reluctant to spend time in intergenerational training [46].

Regarding past sport participation, only 27% declared itself ex-sports. Moreover, who was in the past a former sportsman maintains the level of motor activity indicated by the WHO. Past sport participation was found to be associated with current activity and fitness in this elderly population [47,48]. However, the trend of the responses of this subgroup was unexpected. The more accustomed to weekly PA and the “ex-sport people” were more reluctant toward intergenerational programs than those who are less active. Probably weekly PA performers are already satisfied with their PA and less interested in the intergenerational proposal, although new and unusual PA. A previous study showed that adherence to a PA program is not affected by the participants’ fitness level [49].

Significant inverse correlation between age and the enjoyment for PA were found. Older age was characterized by a sedentary lifestyle, as a reduction of time spent in PA [46], conversely, the lack of correlation between age and enjoyment for intergenerational PA, showed a willingness to this new proposal, regardless of age. Despite elderly usually showed little inclination to dialogue and sharing with younger generations [22], the adherence was motivated by the participation with their family members [50].

The multichoice questions showed that about 80% of the responders should adhere to an intergenerational program. The main benefits that they expect from this activity were that the joint activity may gratify and help them achieve generalized well-being.

Participants preferred a recreational activity based on games, followed by sport activity, which involves both generations in achieving a common goals respect to fitness programs.

Regarding the barriers in carrying out this program, the responders indicated as the main barrier was the fear of fatigue during the activity and the difficulty to keep up with children [51]. These barriers were already recognized as common in elderly in the project “Ri-generiamoci”, however, they would be easily overcome after a period of contact with children [22].

### Limitations

This study did not analyze the children’s feeling regarding the intergenerational physical activity; further studies could evaluate this aspect using different tools adapted for their age.

## 5. Conclusions

The purpose of this study was to provide a basis for developing an intergenerational PA project between preschoolers and elderly, ensuring that it could be a pleasant method to prevent sedentary behaviors typical of older age.

Intergenerational proposals should be more familiar to older Italian people, who are still unaware of their existence, although these programs have for a long time demonstrated benefits for both younger and older people.

To reach success, the PA programs should emphasize the aspect of fun and enjoyment [52]. This survey could represent a stimulus for the organization of intergenerational physical activity programs among children and elderly subjects This survey could represent an approach to organize an intergenerational proposal, based on PA shared between children and older people.

## Figures and Tables

**Table 1:** Sample characteristics.

Variable	*n* (%)
Age (Mean ± SD)	67.8 ± 9.1
<70	76 (54.3%)
≥70	64 (45.7%)
Gender	
Male	48 (34.3%)
Female	92 (65.7%)
Marital status	
Married	86 (61.4%)
Not married	54 (38.6%)
Education	
Elementary and middle school diploma	70 (50.0%)
Secondary school and bachelor degree	70 (50.0%)
Employment	
Housewife and Pensioners	42 (30.0%)
Other job	98 (70.0%)
Residential location	
Rural	42 (30.0%)
Urban	98 (70.0%)
Sport background	
Former-sportsman	38 (27.1%)
Non-sportsman	102 (72.9%)
Sedentariness level	
Active	30 (21.4%)
Inactive	110 (78.6%)
Total sedentary time (Mean ± SD)	469.2 ± 328.5

**Table 2:** PACES-Q and PACES-INT results of the sample subgroups (mean ± SD).

Variable	PACES-Q	PACES-INT
Age		
Under 70	22.55 ± 7.78	15.00 ± 5.44
Over 70	20.19 ± 6.77	16.16 ± 6.46
Gender		
Male	22.29 ± 7.90	17.33 ± 6.47
Female	21.04 ± 7.14	14.59 ± 5.44 #
Marital status		
Married	21.51 ± 7.32	15.05 ± 5.61
Not married	21.41 ± 7.60	16.30 ± 6.39
Education		
Elementary and middle school diploma	21.46 ± 7.00	14.94 ± 5.32
Secondary school and bachelor degree	21.48 ± 7.85	16.11 ± 6.48
Employment		
Housewife and Pensioners	20.09 ± 7.77	13.33 ± 5.32 #
Other job	22.06 ± 7.21	16.47 ± 5.96
Residential location		
Rural	22.19 ± 7.56	12.67 ± 2.62 #
Urban	21.16 ± 7.36	16.76 ± 6.52
Sport background		
Former-sportsman	22.16 ± 7.56	16.10 ± 6.38
Non-sportsman	21.22 ± 7.37	15.31 ± 5.78
Sedentariness level		
Active	19.80 ± 7.91	16.00 ± 6.90
Inactive	21.93 ± 7.24	15.40 ± 5.68

#: significantly differences for PACES-INT.

**Table 3:** Sample subgroups’ enjoyment (%).

Variable	PACES-Q			PACES-INT		
	Positive	Neutral	Negative	Positive	Neutral	Negative
Age						
Under 70	36.8%	18.4%	44.8%	84.2%	10.5%	5.3%
Over 70	53.1%	21.9%	25.0%	78.1%	12.5%	9.4%
Gender						
Male	37.5%	16.7%	45.8%	66.7%	20.8%	12.5%
Female	47.8%	21.8%	30.4%	89.1%	6.5%	4.3%
Marital status						
Married	41.8%	25.6%	32.6%	83.7%	9.3%	7.0%
Not married	48.2%	11.1%	40.7%	78.8%	14.8%	7.4%
Education						
Elementary and middle school diploma	42.9%	25.7%	31.4%	91.4%	2.9%	5.7%
Secondary school and bachelor degree	45.7%	14.3%	40.0%	71.4%	20.0%	8.6%
Employment						
Housewife and Pensioners	52.4%	19.0%	28.6%	95.2%	0%	4.8%
Other job	40.8%	20.4%	38.8%	75.5%	16.3%	8.2%
Residential location						
Rural	42.8%	28.6%	28.6%	100%	0%	0%
Urban	44.9%	16.3%	38.8%	73.5%	16.3%	10.2%
Sport background						
Former-sportsman	36.8%	15.8%	47.4%	78.9%	10.5%	10.5%
Non-sportsman	47.0%	21.6%	31.4%	82.4%	11.8%	5.9%
Sedentariness level						
Active	53.3%	13.3%	33.4%	73.3%	20.0%	6.7%
Inactive	41.8%	21.8%	36.4%	83.6%	9.1%	7.3%

**Table 4:** Multichoice questions on intergenerational program preferences.

Questions	Answers	%
Would you adhere personally to this program?	Yes	78%
	I don’t know	20%
	No	2%
Which kind of activity do you prefer to perform in the intergenerational programs?	Gaming activity	67%
	Sport activity	21%
	Fitness activity	12%
Which benefits would you expect by intergenerational activity?	Generalized well-being	46%
	Social interaction with my grandchild	21%
	Amusement	33%
Which barriers do you think you can meet in performing the intergenerational activity?	I never feel good enough	48%
	Inability to relate to these new generations	15%
	Logistic and family relational problems	37%

## Data Availability

The data that support the findings of this study are available from the corresponding author upon reasonable request.
